# Monitoring of Plasma EGFR Mutations during Osimertinib Treatment for NSCLC Patients with Acquired T790M Mutation

**DOI:** 10.3390/cancers15174231

**Published:** 2023-08-24

**Authors:** Kana Watanabe, Ryota Saito, Eisaku Miyauchi, Hiromi Nagashima, Atsushi Nakamura, Shunichi Sugawara, Nobuyuki Tanaka, Hiroshi Terasaki, Tatsuro Fukuhara, Makoto Maemondo

**Affiliations:** 1 Department of Respiratory Medicine, Miyagi Cancer Center, Natori 981-1293, Japan; watanabe-ka750@miyagi-pho.jp (K.W.);; 2 Department of Respiratory Medicine, Tohoku University Graduate School of Medicine, Sendai 980-8575, Japan; 3 Division of Pulmonary Medicine, Iwate Medical University Graduate School of Medicine, Iwate 028-3895, Japan; 4 Department of Pulmonary Medicine, Sendai Kousei Hospital, Sendai 980-0873, Japan; 5 Division of Cancer Biology and Therapeutics, Miyagi Cancer Center Research Institute, Natori 981-1293, Japan; 6 Molecular Genetic Research Department, LSI Medience Corporation, Tokyo 174-8555, Japan; 7 Division of Pulmonary Medicine, Department of Medicine, Jichi Medical University, Tochigi 329-0498, Japan

**Keywords:** EGFR mutations, T790M mutation, liquid biopsy, sensitive PNA-LNA clamp method, osimertinib

## Abstract

**Simple Summary:**

The sensitive PNA-LNA clamp method could highly detect EGFR gene mutations in plasma. Plasma clearance of the activating gene mutation and the T790M mutation was observed in more than 70% of patients treated with osimertinib, and its clearance was correlated with the efficacy of osimertinib treatment. The C797S mutation, an osimertinib resistance mutation, was detected in only 8.1% of osimertinib-resistant cases.

**Abstract:**

Background: Osimertinib was first approved for the treatment of non-small cell lung cancer (NSCLC) in patients who have developed the epidermal growth factor receptor (EGFR) T790M mutation after treatment with EGFR tyrosine kinase inhibitors (TKIs). We routinely evaluated the plasma of NSCLC patients with the T790M mutation to more rapidly detect an increase in disease activity and resistance to treatment. Methods: Eligible patients received osimertinib after resistance to the first- or second-generation of EGFR-TKIs in NSCLC harboring T790M mutation detectable in tumor tissue or plasma. Plasma samples were collected every 8 weeks during osimertinib treatment. The plasma analysis was performed using an improved PNA-LNA PCR clamp method. We tested samples for a resistance mechanism, including EGFR-activating, T790M, and C797S mutations, and assessed the association between the mutations and osimertinib treatment. Results: Of the 60 patients enrolled in the study, 58 were eligible for this analysis. In plasma collected before osimertinib treatment, activating mutations were detected in 47 of 58 patients (81.0%) and T790M was detected in 44 patients (75.9%). Activating mutations were cleared in 60.9% (28/46) and T790M was cleared in 93.0% (40/43). Of these, 71.4% (20/28) of activating mutations and 87.5% (35/40) of T790M mutation were cleared within 8 weeks of treatment. The total response rate (RR) was 53.4% (31/58). The median duration of treatment was 259 days, with a trend toward longer treatment duration in patients who experienced the clearance of activating mutations with osimertinib. At the time of disease progression during osimertinib treatment, C797S was detected in 3 of 37 patients (8.1%). Conclusion: Plasma EGFR mutation analysis was effective in predicting the effect of osimertinib treatment.

## 1. Introduction

Osimertinib, a third-generation EGFR tyrosine kinase inhibitor (TKI), is the standard of care for the first-line treatment of advanced non-small cell lung cancer (NSCLC) with EGFR-activating mutations and is initially approved for the treatment of NSCLC in patients who develop the EGFR T790M mutation after treatment with a first- or second-generation EGFR-TKIs [1]. The EGFR T790M mutation is the mechanism of resistance in approximately 50–60% of first- and second-generation EGFR-TKIs [2]. In the AURA and AURA2 studies, osimertinib showed high anti-cancer activity in 61–70% of T790M-positive tumors treated with one or more other EGFR-TKIs [3,4,5]. However, osimertinib treatments failed in 10 months on median average after the start of the treatments [6]. There are a variety of changes in resistance mechanisms in patients who experienced progression of the disease while on osimertinib treatments and these changes are utilized in important clinical assessments for subsequent treatment strategies [7]. Tissue biopsies are recommended to confirm gene abnormalities related to resistance, but the biopsies may not be possible due to the small tumor size or location of primary or metastatic sites. On the other hand, liquid biopsy is a less burdensome and simple method. Furthermore, liquid biopsy can be repeated without difficulty [8,9].

Recent studies suggested that clearance of EGFR mutations in plasma after the start of first-line EGFR-TKI treatment predicts response to first- and second-generation TKIs [10,11]. We have previously reported similar results by monitoring plasma EGFR mutations during first-line TKI treatment [12,13].

To evaluate the potential of liquid biopsy in predicting the efficacy of osimertinib, a multicenter prospective observational study was conducted in patients having NSCLC with both EGFR-activating mutations and a T790M mutation. Plasma samples were collected longitudinally during osimertinib treatment until disease progression and then analyzed for gene abnormalities by using PNA-LNA PCR clamp method. In addition, resistance-related mutations were further examined using next-generation sequencing (NGS).

## 2. Materials and Methods

### 2.1. Patients and Study Design

This is a multicenter prospective observational study conducted at four institutions in Japan. In this study, patients had been recruited from December 2016 to December 2019. Eligible criteria were resistance to first- or second-generation EGFR-TKIs and the detection of both activating mutations and a T790M mutation in tumor tissue or plasma. Fifty-eight patients received osimertinib treatment with 80 mg of osimertinib once a day. Plasma samples were collected before treatment and every 8 weeks until failure of osimertinib treatment. The plasma ctDNA analysis was performed using the PNA-LNA PCR clamp method. Samples were tested for EGFR mutations including activating mutations, T790M mutation, and C797S mutation to evaluate the association between EGFR mutations and the efficacy of osimertinib treatment. Furthermore, 22 specimens were analyzed for resistance related to mutations by NGS.

### 2.2. Plasma Sample Collection and EGFR Mutation Analysis 

Blood samples were collected in ethylenediaminetetraacetic acid (EDTA) tubes before TKI administration (P0), every 8 weeks during osimertinib administration (P1), and after disease progression (P2). Samples were well mixed, and plasma separated by centrifugation at 2000 G for 10 min was stored at −20 °C. DNA was then extracted from plasma specimens with QIAamp Circulating Nucleic Acid (QIAGEN, Hilden, Germany). Plasma ctDNA analysis was carried out at the Central Laboratory of LSI Medience Corporation (Tokyo, Japan) using the PNA-LNA PCR clamp method; PCR primers were specifically designed to amplify G719X, exon 19 deletion, T790M, L858R, and L861Q. LNA probes complementary to each mutant allele were generated, and PNA clamps complementary to each wild-type allele were constructed [14,15]. This improved PNA-LNA clamp method used smaller PCR products and increased the number of cycles from 45 to 50 using the Light Cycler 480 Instrument (Roche) to achieve a detection rate of less than 0.1%.

### 2.3. Next-Generation Sequencing Analysis

Next-generation sequencing (NGS) analysis of circulating tumor DNA (ctDNA) in plasma was performed using the AVENIO ctDNA Expanded Kit at the start of osimertinib treatment and at the time of clinical resistance.The AVENIO ctDNA Expanded Kit consists of a next-generation sequencing (NGS) liquid biopsy assay and contains a 77-gene panel that includes genes from the National Comprehensive Cancer Network (NCCN) guidelines and other emerging cancer biomarkers.The Expanded Kit is a pan-cancer assay specifically optimized for lung and colorectal cancer. According to performance data, sensitivity is >96–99% and positive predictive value (PPV) is >98–99% for all four classes (SNV, indel, fusion, and CNV).

### 2.4. Statistical Analysis

Descriptive statistics were applied to evaluate patients’ and mutation characteristics. The time from osimertinib initiation to treatment termination (TTD) was focused on, given that a significant proportion of patients will continue TKI treatment after disease progression due to the clinical benefit. Each incidence of mutation was analyzed using Fisher’s exact test. Survival curves for categorical variables were estimated using the Kaplan–Meier method and compared using the log-rank test; *p* < 0.05 was considered statistically significant. Response Evaluation Criteria in Solid Tumors version 1.1 was used to assess the treatment effect, and all analyses were performed using SPSS version 12 (IBM SPSS Statistics, IBM, Tokyo, Japan).

## 3. Results

### 3.1. Patients

**Figure 1:**
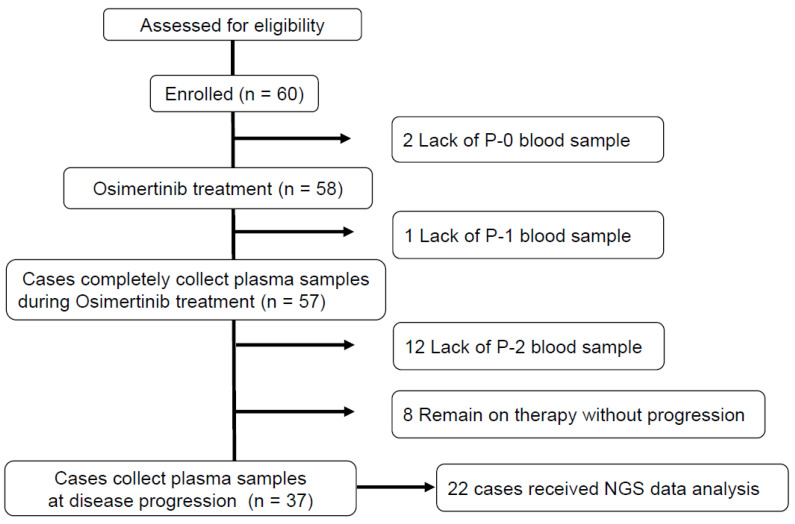
Flow diagram of the eligible study population. Of 60 eligible patients, 58 received osimertinib treatment. P0: Plasma sample before osimertinib treatment, P1: Plasma sample during osimertinib treatment, P2: Plasma sample after disease progression.

Between December 2016 and December 2019, 60 patients with T790M mutation detected in tumor tissue or plasma were enrolled and 2 of whom were excluded from the study due to lack of P0 plasma samples (Figure 1). The clinical characteristics of the subjects were shown in Table 1. The median age was 68 years (range, 43–91), and all had an exon 19 deletion or L858R as the activating mutation of EGFR at diagnosis. One patient harbored a de novo T790M mutation together with L858R at diagnosis. Forty-eight patients (83%) had clinical stage IV metastatic disease at diagnosis, eight (14%) had a postoperative recurrence, and two (3%) had a post-chemoradiotherapy recurrence. Twenty-six (45%) had T790M mutation confirmed by tissue re-biopsy, 6 (10%) by pleural effusion, and twenty-six (45%) by liquid biopsy. Before enrollment, T790M was analyzed by Cobas in 35 cases and by PNA-LNA PCR Clamp in 23 cases.

### 3.2. Plasma EGFR Mutations during Osimertinib Treatment and at Progression

**Figure 2:**
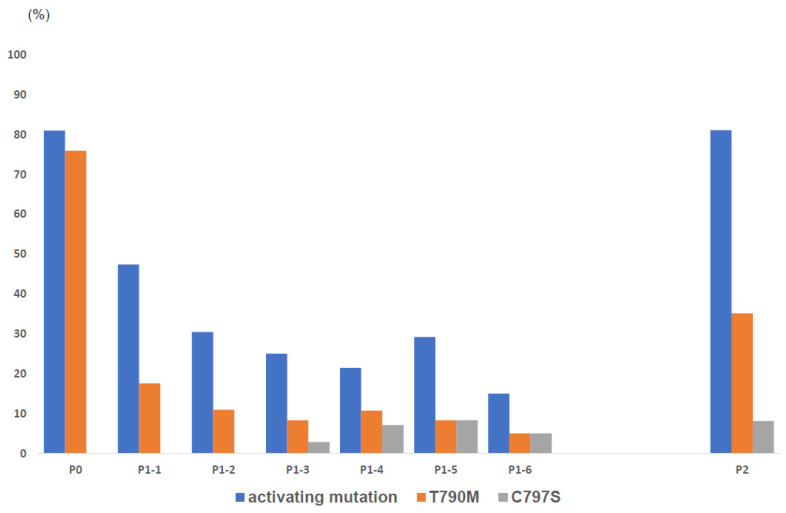
Frequency of EGFR mutations in plasma at each blood collection time point. The frequency of T790M mutation, C797S, and EGFR-activating mutations of exon 19 deletion and L858R were shown. Mutations in plasma were evaluated by using the improved PNA-LNA PCR clamp method before osimertinib treatment (P0), every 8 weeks during osimertinib treatment (P1-), and after progression of disease (P2). Results after P1–7 were omitted due to the small number of specimens.

The EGFR mutation status in plasma was analyzed by using the PNA-LNA PCR clamp method and the results are shown in Table 2. We evaluated the mutations in plasma every 8 weeks of osimertinib treatment. The frequency of EGFR mutations detected in plasma at each time point was shown in Figure 2. At baseline, the detection rate of EGFR-activating mutations and T790M mutation were 81.0% and 75.9%, respectively. These frequencies were dynamically changing, with a marked decrease at 8–16 weeks and an increase during disease progression (PD). In the 57 patients with available plasma samples during treatment, activating mutations were cleared in 60.9% (28/46), and the T790M mutation was cleared in 93.0% (40/43). Of these, 71.4% (20/28) of activating mutations and 87.5% (35/40) of T790M mutations were cleared within 8 weeks after the start of osimertinib treatment. Forty-nine patients experienced PD during osimertinib treatment. EGFR mutation status was able to be analyzed for 37 of 49 patients having PD. Among the 37 patients, activating mutations were detected in 30 (81.1%) and T790M mutations in only 13 (35.1%). The incidence of C797S during osimertinib treatment was 8.1% (3/37).

### 3.3. Efficacy and Plasma EGFR Mutation Status of Osimertinib Treatment in Patients with EGFR T790M Mutation

One patient achieved complete response (CR), thirty patients had partial response (PR), and twenty-three patients had stable disease (SD). The objective response rate (ORR) was 53.4%. When comparing those who were positive for activation mutation at P0 to those who were negative, those who were negative had a higher response rate compared to those who were positive (81.8% vs. 46.8%). There was no difference between the two for T790M (positive: 54.5% vs. negative: 50.0%) (Table 3). The relationship between plasma EGFR mutation analysis and the duration of osimertinib treatment is shown in Supplementary Figure S1. Patients with high response rates tended to achieve long periods of osimertinib treatment. 

**Figure 3:**
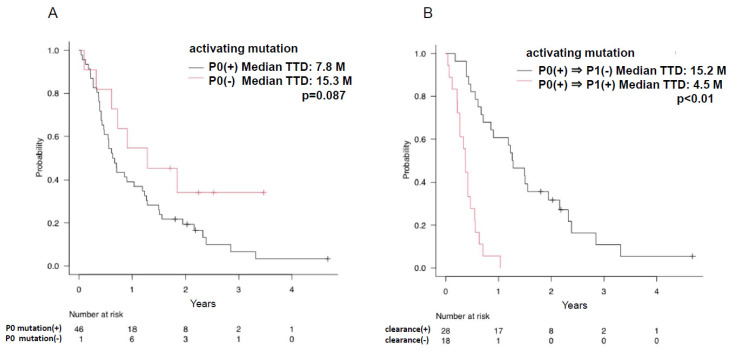
Relationship of detectable activating mutation and time to treatment discontinuation (TTD). (**A**) The time to discontinuation of therapy (TTD) is shown by the Kaplan–Meier curve for each patient with or without activating mutations detected in plasma prior to osimertinib treatment. Patients without EGFR-activating mutations are shown by the red line and patients with EGFR mutation are shown by the black line. (**B**) Kaplan–Meier curves for TTD are shown according to whether activating mutations detected in plasma before osimertinib treatment disappeared after 8 weeks; patients with residual EGFR-activating mutations are indicated by red lines and cases that have disappeared are shown as black lines.

**Figure 4:**
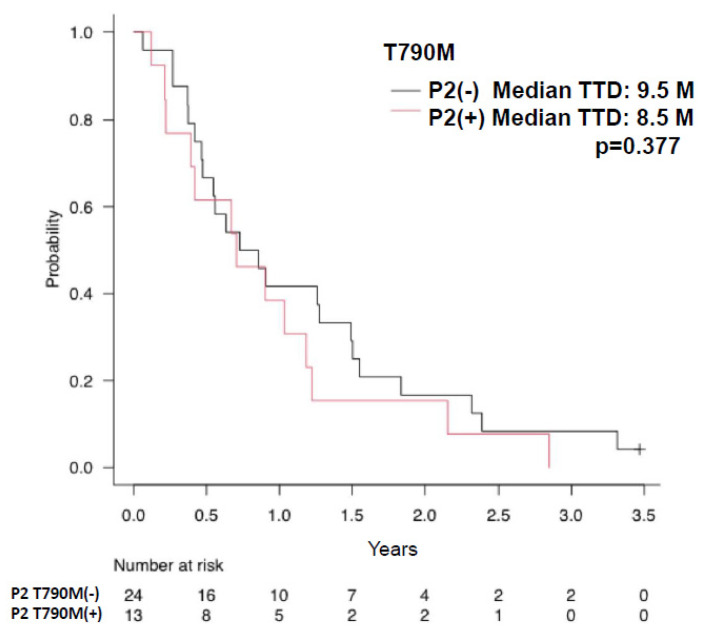
Relationship of appearance of T790M mutation after osimertinib treatment and time to treatment discontinuation (TTD). Kaplan–Meier curves of TTD are shown according to the appearance of T790M when the patients became resistant to osimertinib treatment (P2). Patients with positive T790M mutation at the time of osimertinib resistance are indicated by red lines, and patients in whom the T790M mutation did not appear are indicated by black lines.

The median TTD (time to treatment discontinuation) was longer for patients with no EGFR-activating mutations detected in plasma at baseline than for those with EGFR mutations (15.3 months vs. 7.8 months, respectively, *p* = 0.087) (Figure 3A). Patients with plasma clearance of activating EGFR mutations at 8 weeks after initiation of treatment had a significantly longer median TTD than those without clearance of EGFR mutations (15.2 months vs. 4.5 months, respectively, *p* < 0.01) (Figure 3B). There was no difference in TTD between cases with and without detection of T790M at PD (P2) (9.5 months vs. 8.5 months, respectively, *p* = 0.377) (Figure 4).

### 3.4. NGS Analysis of Plasma before and after Osimertinib Treatment

**Figure 5:**
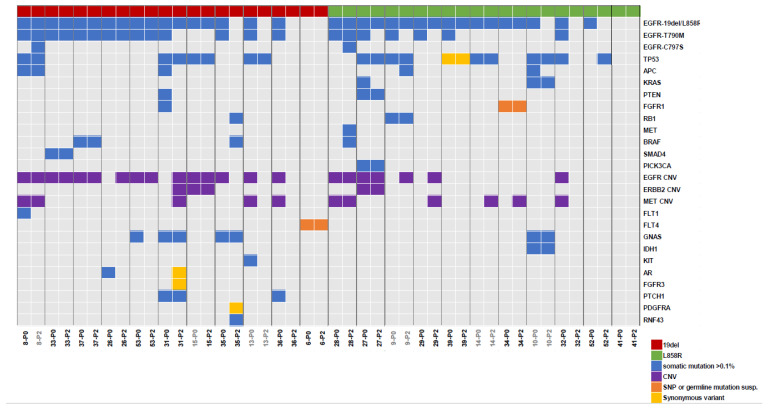
Gene mutation profiles by next-generation sequencing before treatment, with osimertinib (P0) and after disease progression (P2). P0 and P2 are shown side by side. The attached numbers are sample numbers. Color coding is shown for each type of genetic abnormality.

Abnormalities in somatic genes in plasma before osimertinib treatment and at PD were examined by NGS; a total of 22 patients were available for NGS analysis (Figure 5). The total number of somatic alterations detected in cfDNA was 75 in pretreatment samples and 63 in samples at disease progression. EGFR-activating mutations were detected in the cfDNA of 20 (90.9%) of the 22 pretreatment plasma samples, and the type of activating mutation was the same as that identified in tumor tissue or plasma before study enrollment. The EGFR T790M mutation was detected in 15 (68.2%) of 22 pretreatment plasma samples of cfDNA, but the detection rate from using NGS was less frequent than from using the PNA-LNA PCR clamp method. The detection rate for activating mutations of EGFR and T790M in cfDNA using NGS at disease progression was lower than before treatment. TP53 was the most common gene mutation other than EGFR, found in 40.9% of patients both before treatment and after progression. EGFR C797S mutation and MET gene abnormalities were identified only after disease progression. 

## 4. Discussion

This study aimed to investigate the role of plasma monitoring during osimertinib treatment in patients with T790M-positive advanced NSCLC refractory to prior EGFR-TKIs.

In this study, the plasma ctDNA analysis was performed using an improved PNA-LNA PCR clamp method. The frequency of plasma-activating mutations before study treatment (P0) was 81.0%, and T790M was 75.9%, indicating that the PNA-LNA clamp method has adequate capacity as a liquid biopsy technique. Undetected EGFR mutations before treatment and the clearance of EGFR mutations in liquid biopsy were shown to be predictive of treatment benefit.

Currently, NGS is often used for liquid biopsies because it can measure numerous genes at once, but its high cost makes it unsuitable for monitoring because it requires multiple measurements. In the case of monitoring, it is unavoidable to measure a limited number of genes by PCR. In this context, the PCR-based Cobas assay is contrasted with the PNA-LNA clamp method used in this study. The semiquantitative PCR-based Cobas assay (Roche Molecular Systems, Pleasanton, CA, USA) is the only plasma genotyping assay currently approved by the FDA; approval of this assay for the detection of EGFR sensitizing mutations was based on a post-hoc analysis in the ENSURE study. Both plasma and tumor tissue were tested using the Cobas assay with a sensitivity of 76.7% (range 70.5–81.9%) for the detection of EGFR-sensitizing mutations and a specificity of 98.2% (range 95.4–99.3%) when using tissue genotypes as reference standards [16]. This approval was later extended to detect the T790M acquired resistance mutation of the EGFR in phase II studies of osimertinib in EGFR-mutant NSCLC with acquired resistance to kinase inhibitors (AURA extension; NCT0180-2632 and AURA2; NCT02094261) using plasma and tissue pairs collected from patients with EGFR-mutant NSCLC. Results of this analysis, sensitivity, and specificity for detecting EGFR T790M were 93% and 92% [17]. 

The improved PNA-LNA PCR clamp method can achieve a high detection rate of EGFR mutations at a low cost. The original PNA-LNA PCR clamp method is commercially available in Japan, but its sensitivity is about 1%. We improved the sensitivity to 0.1% by modifying the primer sites and the thermal cycler. This method has an advantage over dPCR and NGS in terms of cost-benefit ratio. The results of a small trial we examined using the PNA-LNA clamp method were: sensitivity, 79.2%; specificity, 100% [12]. In a direct comparison of the PNA-LNA clamp and Cobas methods in a small study, the performance of the PNA-LNA clamp method was comparable [18]. In this study, NGS was performed on residual liquid samples used in the PNA-LNA clamp method and compared with the PNA-LNA clamp method. In particular, a tendency toward lower detection rates of T790M was observed. We can conclude that the PNA-LNA clamp method is not inferior to NGS, at least in terms of the detection rate of the target.

Using this method, plasma monitoring during osimertinib treatment could be performed. Several studies showed that ctDNA clearance during first-line EGFR TKI treatment predicted the outcome of first- and second-generation TKI treatment [11,19,20]. Ma et al. reported that ctDNA clearance correlated with prolonged PFS and OS for third-generation EGFR TKIs, while T790M levels were not associated with poorer outcomes while using a large genetic NGS panel of 425 genes. These results suggested that clearance of activating mutations in plasma was a positive predictor of prognosis in patients treated with EGFR TKIs [21,22,23]. On the other hand, previous analyses of patients with T790M NSCLC reported that patients with clearance of detectable EGFR T790M had a shorter median time to treatment discontinuation than those with retained EGFR T790M (6.1 months vs. 15.2 months, respectively) [24,25].

In our study, we also found that the median TTD was significantly shorter for patients with detectable EGFR-activating mutations in plasma at baseline than for those without detectable mutations, and the median TTD was significantly longer for patients whose mutations disappeared during treatment than for those who did not.

In clinical practice, diagnosis of disease progression has relied primarily on radiological imaging. However, monitoring EGFR mutations in plasma during TKI treatment may detect disease progression earlier. We observed that EGFR mutation re-detection or re-elevation occurs 6.0 months earlier than the degree of progression displayed on medical imaging (Supplementary Figure S2). This indicates that molecular events occur earlier than clinical changes and this is consistent with previous studies focusing on ctDNA detection in early cancer recurrence [26,27,28].

In the present study, the frequency of C797S as a resistance mechanism for osimertinib was lower than previously reported [25,29]. *TP53* was the most common gene mutation other than EGFR, found in 40.9% of cases. *TP53* mutations are highly correlated with smoking in EGFR-mutant lung cancer, with an incidence of about 50% and are the most common concurrent mutations [30]. Concurrent *TP53* mutations have been shown to be a negative prognostic factor and associated with poorer outcomes in patients treated with EGFR-TKIs [31]. In this study, there was no obvious relationship between *TP53* and TTD (Supplementary Figure S3). 

NGS, which is now frequently used in liquid biopsy, was performed for comparison with the PCR method used in this study. It is not intended to extract new resistance-related genes.

This study has several limitations, including small sample size and the fact that NGS analysis was not performed on all patient samples. We collected and analyzed plasma samples every 8 weeks, but it cannot be determined from this study whether the frequency was accurate. There are many different mechanisms of resistance and predictors of efficacy, including transformation, and not all of them were detected and examined in this study. Clearly, there are areas where tissue samples show an advantage. In the future, it will be important to utilize the advantages of both tissue and liquid samples.

## 5. Conclusions

In conclusion, the cases with undetectable EGFR-activating mutations in plasma prior to treatment tended to have a longer duration of treatment. In addition, clearance of EGFR-activating mutations after the initiation of osimertinib therapy was associated with a favorable prognosis. On the other hand, there was no association between the presence or absence of T790M detection in plasma and the duration of treatment. Monitoring EGFR mutations in plasma may detect relapse earlier than imaging. Liquid biopsy may facilitate early detection of intrinsic or acquired resistance.

## Figures and Tables

**Table 1:** Baseline patient characteristics at the time of osimertinib initiation.

		n	%
Age	Median (range)	68 (43–91)	
Gender	Male	25	43
	Female	33	57
EGFR mutation at daignosis	Del19	32	55
	L858R	25	43
	L858R + de novo T790M	1	2
Disease stage	IIIB/IV	48	83
	Postoperative recurrence	8	14
	Post-chemoradiotherapy recurrence	2	3
Source of T790M	Tissue	26	45
	Pleural effusion	6	10
	Blood	26	45
T790M analysis methods	Cobas	35	60
	PNA-LNA PCR Clamp	23	40
Treatment line	2nd	30	52
	3rd	11	19
	4th+	17	29
First EGFR-TKI	Gefitinib	25	43
	Erlotinib	11	19
	Afatinib	22	38

**Table 2:** Plasma EGFR mutations during osimertinib therapy.

	P0	P1-1	P1-2	P1-3	P1-4	P1-5	P1-6	P2
N	58	57	46	36	28	24	20	37
Activating mutations	47	27	14	9	6	7	3	30
(%)	81.0	47.4	30.4	25.0	21.4	29.2	15.0	81.1
T790M	44	10	5	3	3	2	1	13
(%)	75.9	17.5	10.9	8.3	10.7	8.3	5.0	35.1
C797S				1	2	2	1	3

P0: before osimertinib treatment, P1: during treatment, P2: after progression of disease, P1 samples were collected every 8 weeks. Results after P1–7 were omitted due to the small number of specimens.

**Table 3:** Efficacy of osimertinib treatment—n (%).

	Total	Activating Mutation		T790M	
	n = 58	P0(+) n = 47	P0(−) n = 11	P0(+) n = 44	P0(−) n = 14
CR	1 (1.7)	1 (2.1)	0	1 (2.3)	0
PR	30 (51.5)	21 (44.7)	9 (81.8)	23 (52.3)	7 (50.0)
SD	23 (39.7)	21 (44.7)	2 (18.2)	16 (36.4)	7 (50.0)
PD	2 (3.4)	2 (4.2)	0	2 (4.5)	0
NE	2 (3.4)	2 (4.2)	0	2 (4.5)	0
ORR (%)	53.4	46.8	81.8	54.5	50.0

## Data Availability

The data can be shared up on request.

## Supplementary Materials

The following supporting information can be downloaded at: https://www.mdpi.com/article/10.3390/cancers15174231/s1, Figure S1:Relationship of plasma analysis and duration of osimerinib treatment; Figure S2: Dynamic monitoring and imaging assessment of EGFR mutations in plasma; Figure S3: Relationship of detectable TP53 and time to treatment discontinuation (TTD).
