# Supplementary material for: Monitoring of Plasma EGFR Mutations during Osimertinib Treatment for NSCLC Patients with Acquired T790M Mutation
Source: Cancers (Basel). 2023 Aug 24;15(17):4231. doi: 10.3390/cancers15174231 (PMC10486675; doi:10.3390/cancers15174231)
Supplement: Supplementary file 1 [file cancers-15-04231-s001.zip › cancers-2546066-supplementary.pdf]

Supplementary Figure S1

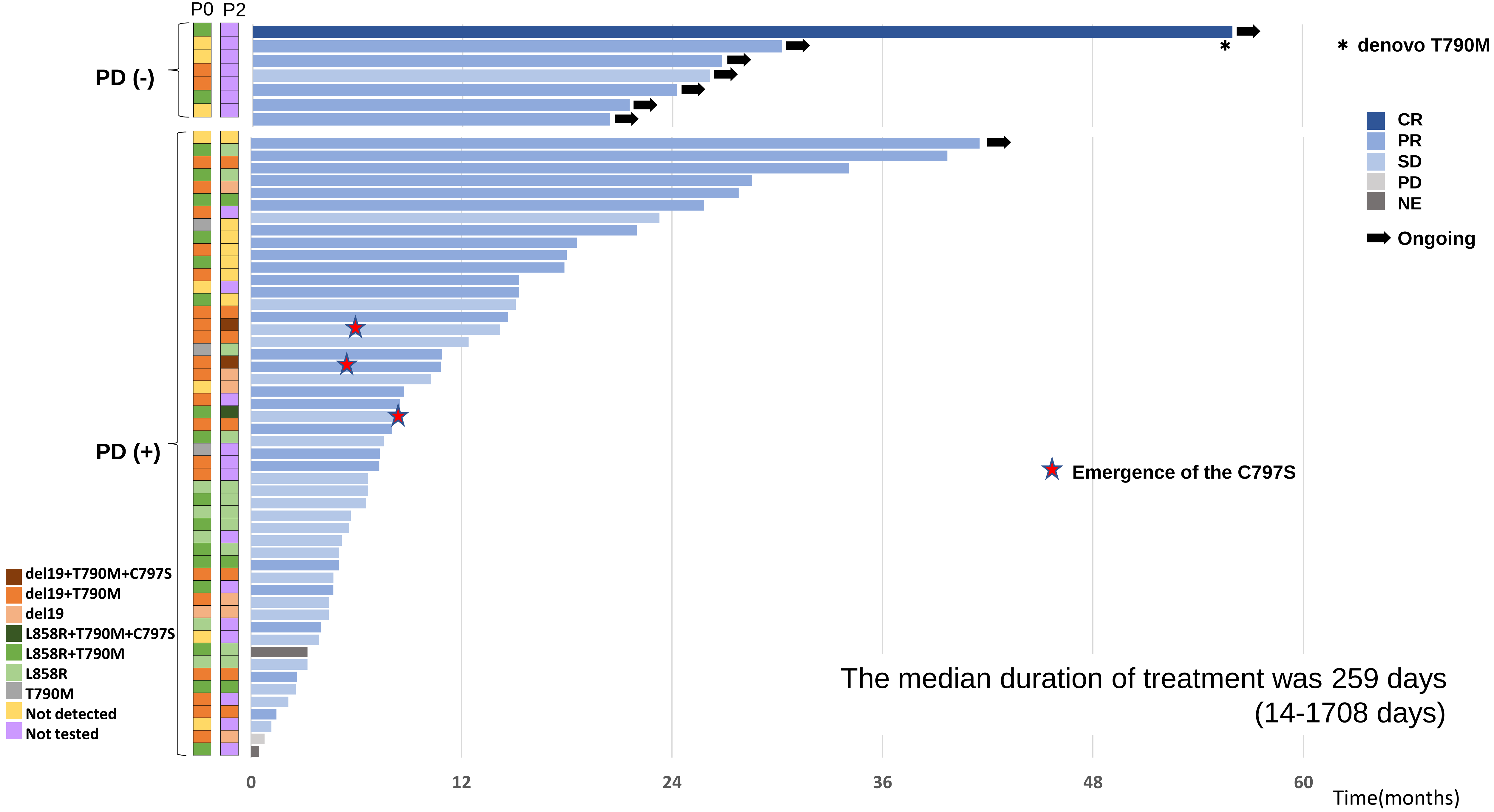

The median duration of treatment was 259 days (14-1708 days)

Supplementary Figure S2

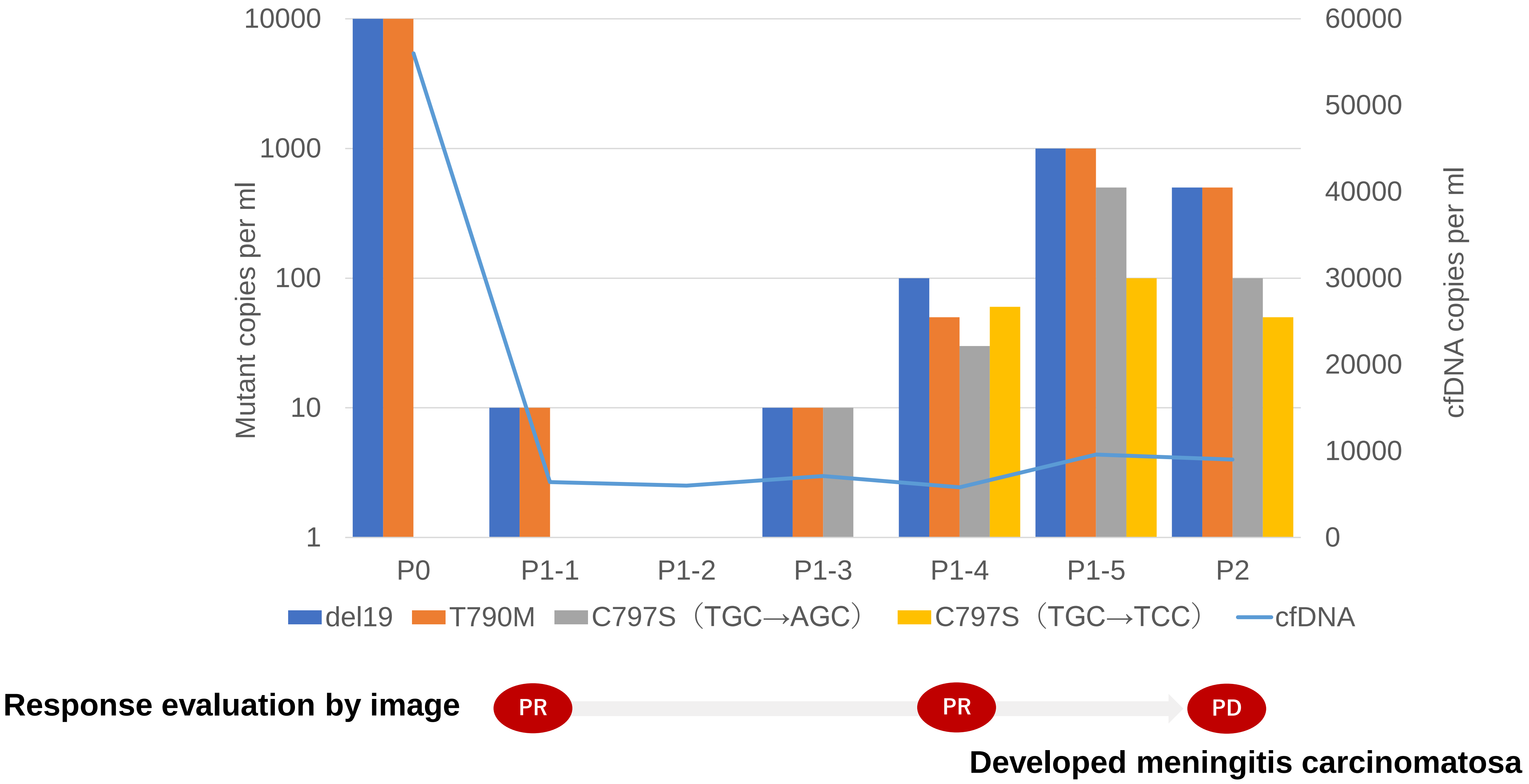

Supplementary Figure S3

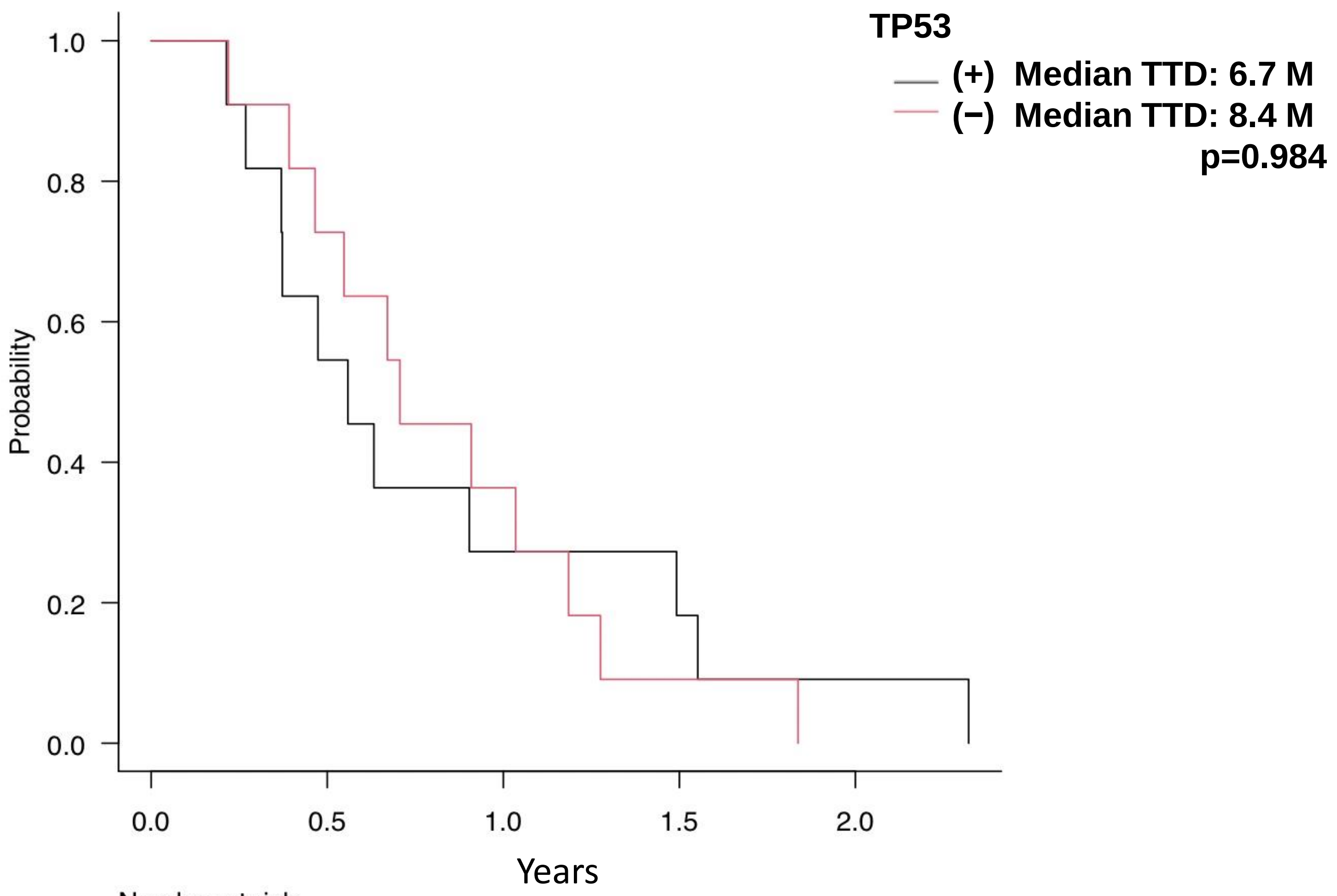

Number at risk

TP53(+)	11	6	3	2	1
TP53(-)	11	8	4	1	0
